# Beneficial Effects of Phytogenic Feed Additives on Epithelial Barrier Integrity in an In Vitro Co-Culture Model of the Piglet Gut

**DOI:** 10.3390/molecules28031026

**Published:** 2023-01-19

**Authors:** Dominik Wendner, Theresa Schott, Elisabeth Mayer, Klaus Teichmann

**Affiliations:** DSM—BIOMIN Research Center, Technopark 1, 3430 Tulln, Austria

**Keywords:** feed additives, phytogenic, plant based, cell culture, in vitro, porcine, co-culture, gut health, intestinal epithelium, animal welfare

## Abstract

Industrial farming of livestock is increasingly focused on high productivity and performance. As a result, concerns are growing regarding the safety of food and feed, and the sustainability involved in their production. Therefore, research in areas such as animal health, welfare, and the effects of feed additives on animals is of significant importance. In this study, an in vitro co-culture model of the piglet gut was used to investigate the effects of two phytogenic feed additives (PFA) with similar compositions. Intestinal porcine epithelial cells (IPEC-J2) were co-cultivated with peripheral blood mononuclear cells (PBMC) to model the complex porcine gut environment in vitro. The effects of treatments on epithelial barrier integrity were assessed by means of transepithelial electrical resistance (TEER) in the presence of an inflammatory challenge. Protective effects of PFA administration were observed, depending on treatment duration and the model compartment. After 48 h, TEER values were significantly increased by 12–13% when extracts of the PFA were applied to the basolateral compartment (*p* < 0.05; n = 4), while no significant effects on cell viability were observed. No significant differences in the activity of a PFA based mainly on pure chemical compounds versus a PFA based mainly on complex, natural essential oils, and extracts were found. Overall, the co-culture model was used successfully to investigate and demonstrate beneficial effects of PFAs on intestinal epithelial barrier function during an inflammatory challenge in vitro. In addition, it demonstrates that the two PFAs are equivalent in effect. This study provides useful insights for further research on porcine gut health status even without invasive in vivo trials.

## 1. Introduction

High performance standards in the modern farming of livestock, such as in the swine industry, come with several challenges, all of which affect the animal’s life, welfare, and healthy growth. These challenges are caused by a variety of stressors such as pathogens associated with the farming environment and the presence of (myco-)toxins or other contaminants in the feed. Additionally, routine rearing processes, such as weaning, challenge the animals and their overall productivity [1]. Particularly, deficiencies and impairments of the gastrointestinal (GI) tract and microbiota, combined with subsequent or simultaneous irregularities of the immunological system, e.g., inflammation, are likely to negatively affect animal health on a systemic level [2,3]. Poor animal gut health also leads to reduced performance, growth, and meat quality [4]. Economic losses due to clinical or subclinical problems are likely consequences [3]. Hence, it must be of utmost interest for individual small-scale farmers, as well as bigger farming conglomerates, and any dependent connected business and industry, to improve animal welfare and health at the farm level and continuously improve feed and feed additives. Phytochemicals and plant-derived feed additives (PFA or so-called phytogenics) [5,6,7,8,9,10,11,12], probiotics [4,13,14], as well as other substances of natural or synthetic origin, and optimized feeding strategies [3] can be used to improve piglet GI health and growth performance during all rearing stages, e.g., during [15,16] and after weaning [17,18,19,20]. Livestock should be enabled to better deal with the numerous challenges of industrial farming, rather than solely rely on reactive treatment of illnesses, underperformance, and poor animal welfare.

PFAs are subject to various in vivo and in vitro studies across species, shedding light on diverse areas of potential activity [5,6,7,11,12,21] such as immunological status and inflammation [16,21,22,23,24,25], gastrointestinal health and barrier integrity [16,22,23,24,25,26,27], protective effects against pathogens and toxins of various kinds [23,24,25,27], as well as overall animal growth and performance [15,21,24,26,27]. In general, suitable in vitro models are of significant importance and many available in vitro studies assess specific subprocesses of gut functionality and interactions. A typical example are the gut barrier integrity models, for which the Caco-2 cell line is often described as “the gold standard”, even for animals, although it is derived from a human colorectal carcinoma [28,29]. However, similar models have also been described using species-specific cell lines, such as IPEC-J2, an intestinal epithelial cell line derived from the jejunum of a neonatal piglet [23,28,30,31,32]. More complex models employ direct or indirect co-culture of different cell types, e.g., epithelial and immune cells, in order to better approximate the multilayers of in vivo gut processes. Nevertheless, many such models do not utilize cell lines derived solely from the target organism, or are focused on human application [33,34,35,36]. Furthermore, a limited number of in vitro trials related to intestinal processes with multiple, co-cultured porcine cell lines are available. These trials usually focus on the investigation of the negative effects of toxins and pathogens on the intestinal epithelium [23,37,38,39]. Recently, a co-culture model of the piglet gut has been described, which utilizes both intestinal porcine epithelial cells from the jejunum of a neonate piglet (IPEC-J2) and peripheral blood mononuclear cells (PBMC) isolated from porcine blood [40]. In this porcine model, the intestinal barrier of the gut is simulated in a way that is more complex und presumably closer to in vivo conditions than the single cell line barrier models, or the use of model cell lines that are not species-specific. This porcine gut model has already been used to test essential oils and extracts for beneficial effects on barrier integrity and gut health, specifically intended for pigs as the target species [40], and could also be suitable for a broad range of other sample types, e.g., nutritional components, drugs, or PFAs.

According to the “Three R” principle for animal welfare [41,42,43], researchers should aim to replace, reduce, and refine animal trials wherever possible. This in vitro model is an interesting tool to aid in achieving that goal. Promising candidates for subsequent in vivo trials can be identified based on parameters such as barrier integrity, anti-inflammatory properties, or effects on cellular viability, and studies on their mechanisms of action can be conducted. The ultimate goal of the study at hand was to use the model described by Schott et al. [40] for the testing of two commercial PFAs, based either mainly on natural essential oils and extracts, or mainly on pure chemical compounds. Both PFA were composed of encapsulated, volatile ingredients, plant powders, and extracts. The primary goal included four aspects: (1) We assessed potential gut barrier protective effects of the two PFAs in the co-culture in vitro model, without the immediate need for invasive in vivo studies. (2) We compared the effects of a PFA based mainly on pure single chemical compounds, with one based mainly on natural complex compound mixtures. (3) We tried to elucidate the PFA’s mechanism of action on the interplay of epithelial barrier function and immune cells, with regards to the effects of treatment time and site of application. (4) We intended to test the suitability of the in vitro co-culture model of the piglet gut for studies of feed additives with complex composition, in addition to the previously studied single plant extracts or pure substances.

## 2. Results

### 2.1. Co-Culture Model—Barrier Integrity

**Figure 1:**
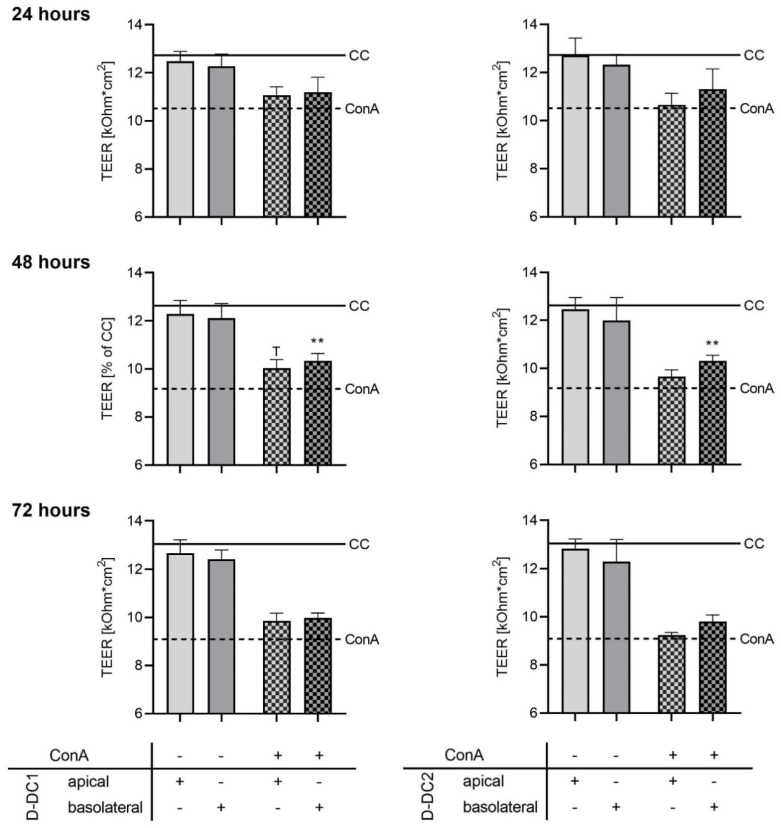
Effects of D-DC1 and D-DC2 extracts (applied at 150 µg/mL) on the co-culture model of the piglet gut, with apical or basolateral application and with or without ConA-stimulation. TEER [kOhm*cm^2^] was observed over a 72 h period. Absolute TEER values are shown compared with the unstimulated CC (solid line). Effects of extracts D-DC1 (on the left) and D-DC2 (on the right) without ConA-stimulation were compared statistically with the unstimulated CC. Effects of the extracts in the presence of ConA-stimulation were compared statistically with the ConA-stimulated control (dashed line, ConA). Bars and error bars represent the means and standard deviations of 4 independent experiments (n = 4). Significant differences are indicated by asterisks ** *p* < 0.01, ^T^ trend, *p* < 0.1).

Effects of 150 µg/mL of the PFA extracts Digestarom^®^ DC 1 (D-DC1) and Digestarom^®^ DC 2 (D-DC2) on epithelial barrier integrity were investigated in the indirect co-culture model in the absence and presence of a ConA challenge (Table 1 and Figure 1). Epithelial barrier integrity (TEER, [kOhm*cm^2^]) was assessed over a 72 h incubation period after the application of the ConA stimulus and extract treatments, and compared with two control groups at each time point: the unstimulated cell control (CC) and stimulated ConA control (ConA). The ConA group showed significantly reduced TEER values compared with the CC group at all time points (*p* < 0.05). Apical (=“api”) and basolateral (=“baso”) extract groups without ConA stimulus did not show a significant change in TEER compared with the CC group after 24, 48, and 72 h (*p* > 0.05). After 24 h, no significant differences compared with the CC group were observed for D-DC1 api + ConA (trend, *p* = 0.0807) and D-DC1 baso + ConA (*p* > 0.1), as well as D-DC2 baso + ConA (*p* > 0.1), while a significant reduction in TEER was observed for D-DC2 api + ConA (*p* = 0.0347). After 48 and 72 h, all groups with extract treatment and ConA stimulus challenge showed significantly reduced TEER values compared with the unstimulated CC (*p* < 0.05).

The CC group and all unstimulated groups with basolateral and apical extract application showed significantly higher TEER values compared with the ConA group at all time points (*p* < 0.05), indicating a successful ConA stimulus challenge to the barrier integrity. Extract treatment of stimulated wells did not have significant effects compared with the ConA group after 24 h, regardless of extract type and application site (*p* > 0.05). After 48 h, significant TEER increases compared with the ConA control group were observed for the groups D-DC1 baso + ConA (+12.6%, *p* = 0.0082) and D-DC2 baso + ConA (+12.4%, *p* = 0.0046). No significant effects were observed for the groups D-DC2 api + ConA (+5.3%, *p* > 0.05) and D-DC2 api + ConA (+9.4%, trend, *p* = 0.0507). After 72 h, no significant effects compared with the ConA control were observed for the extract treatment groups with the ConA stimulus challenge. Repeated measures two-way ANOVA showed significant effects of time and treatment alone, and the interactions of treatment type and incubation time (Appendix B, Table A1, *p* < 0.0001). The matching of trial replica wells (repeated measures over 24, 48, and 72 h) was effective and is considered significant (*p* = 0.0014). However, treatment effects accounted for the vast majority of the observed variation of data (79.1%).

### 2.2. Co-Culture Model—Cell Viability

**Figure 2:**
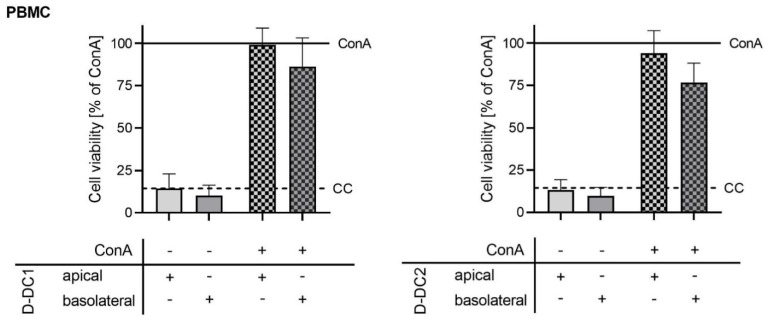
Effects of D-DC1 (**left**) and D-DC2 (**right**) extracts (150 µg/mL) on the viability of PBMC in the co-culture model. The viability of PBMC was determined after the conclusion of barrier integrity tests, 72 h after the application of stimuli and treatments. Bars and error bars represent the means and standard deviations of 4 independent trials (n = 4). Effects of extracts D-DC1 (on the left) and D-DC2 (on the right) without ConA-stimulation were compared statistically with the unstimulated CC (dashed line). Effects of the extracts in the presence of ConA-stimulation were compared statistically with the ConA-stimulated control (solid line, ConA), as a measure of a fully activated, stimulated PBMC population (100%).

**Figure 3:**
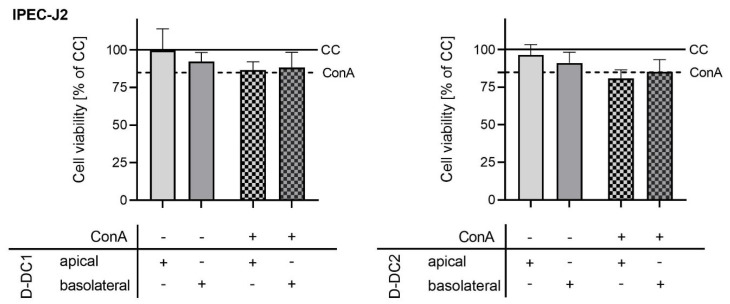
Effects of D-DC1 (**left**) and D-DC2 (**right**) extracts (150 µg/mL) on the viability of IPEC-J2 in the co-culture model. The viability of IPEC-J2 was determined after barrier integrity tests, 72 h after the application of stimuli and treatments, by Neutral Red (NR) test. Bars and error bars represent the means and standard deviations of 4 independent trials (n = 4). Effects of extracts D-DC1 (on the left) and D-DC2 (on the right) without ConA-stimulation were compared statistically with the unstimulated CC (solid line, 100%). Effects of the extracts in the presence of ConA-stimulation were compared statistically with the ConA-stimulated control (dashed line, ConA).

Cell viability testing was carried out at the end of the 72 h TEER observation period, in order to account for possible effects of the stimulus and/or test substances on the viability of PBMC (Figure 2) and/or IPEC-J2 (Figure 3). Relative PBMC viability was calculated with the ConA group set to 100% (Figure 2). Data showed no significant differences in cell viability when comparing treatment groups with the CC group (*p* > 0.05). Comparison of cell viability with the ConA group showed significant differences for the non-stimulated extract groups (*p* < 0.05) and a numerical trend for the CC group (*p* = 0.076). None of the stimulated extract groups showed significant differences compared with the ConA group (*p* > 0.05).

Additionally, the effects of the PBMC activator ConA and/or the application of product extracts on the viability of IPEC-J2 were assessed using the Neutral Red (NR) uptake test. Relative IPEC-J2 cell viability was calculated with the CC group set to 100% and statistical analysis was carried out. None of the non-stimulated extract groups showed significant differences in viability compared with the CC group (*p* > 0.05). The ConA group showed a trend of reduced cell viability by 15.2% (*p* = 0.0738). Basolateral and apical addition of the extract D-DC1 in addition to the stimulus did not reduce IPEC-J2 viability significantly (*p* > 0.05). A significant reduction of IPEC-J2 cell viability by 19.2% was observed for the D-DC2 api + ConA group (*p* = 0.0143), while a numerical viability reduction by 14.8% in the D-DC2 baso + ConA group was not significant (trend, *p* = 0.0859). Cell viability in stimulated product groups, basolateral or apical, did not differ significantly from the ConA stimulation control group (*p* > 0.05).

## 3. Discussion

### 3.1. Effects of Phytogenic Products, Apical or Basolateral Application, and Treatment Time on the In Vitro Co-Culture Model

A novel, complex, in vitro co-culture model of the piglet gut that has been used before for the assessment of potential barrier protective effects of single phytogenic compounds, such as oregano oil or licorice extract [40], was successfully used to evaluate two complex PFAs. It consists of an apical, or luminal, side with an IPEC-J2 layer, corresponding to the intestinal lumen of the live animal, and a basolateral compartment with PBMC, representing the inner tissue and blood vessels of the animal. An inflammation-induced disruption of IPEC-J2 barrier integrity, due to activation of basolateral PBMC, is simulated by stimulation with ConA. Potential mitigation by treatment with test substances is observed, using TEER as a parameter for barrier integrity in the gut. In our study, an effective ConA-induced challenge was observed, indicated by significantly reduced barrier integrity in the ConA control over the entire 72 h trial period. Major research questions were related to the effects of two PFAs in the model, their chemical compositions, possible influences of the targeted application site (apical or basolateral), incubation and treatment times, and PBMC populations in order to elucidate their possible mode of action. “Treatment type”, i.e., the respective chosen product and application compartment, with and without ConA stimulus, was identified as the largest contributor to significant data variation in the study at hand. Additionally, the specific incubation time was identified as a significant contributor, indicating time-dependent effects of PFA extract treatments, as well as significant interactions of time and treatment effects.

At the initial measurement after 24 h, the ConA stimulus control and the treatment group with apical D-DC2 application, in addition to ConA stimulus, showed significantly reduced TEER compared with the CC. The absence of significant TEER reductions in ConA-stimulated groups with test products indicates slower or less severe effects of the ConA stimulus on barrier integrity in these groups (i.e., both D-DC1 treatments, as well as basolateral D-DC2) at this specific timepoint. At a time of 48 h after stimulus, all ConA-stimulated groups showed TEER values significantly lower than the CC. At the same time, significantly higher TEER values than in the ConA control group were observed in both basolateral treatment groups. Results indicate significant beneficial, barrier-protective effects of basolateral application. Direct exposure of PBMC to both PFAs had a larger effect on the test system than direct exposure of IPEC-J2 in the apical compartment, as only trends for barrier protection could be observed for apical extract application. This may be explained by the anti-inflammatory effects of active compounds [20,25,40,44,45,46] directly on site, counteracting the inflammation reaction and barrier integrity challenge simulated by activation of basolateral PBMC by ConA. After 72 h of incubation, no significant effects or trends compared with the ConA control could be observed in all treatment groups, indicating reduced efficacy of treatments over time. Overall, both PFAs showed significant barrier protective effects upon basolateral addition to the test system, building up beneficial effects over the first 48 h of incubation. Optimal effects were observed 48 h after stimulation and the start of treatments, before receding again at the last measurement point.

In an in vivo setting, cells are constantly proliferating, and damaged or aged cells undergo apoptosis, are replaced, and are continually provided with necessary fresh nutrients. Similarly, in vitro test systems, such as the one at hand, are subject to the same effects. When nutrients and media cannot be replenished or active compounds of the test samples are degraded or metabolized over time, observed beneficial effects may be reduced and viability, proliferation, and overall properties of cell populations are adversely affected, with activated inflammatory pathways, cell death, or apoptosis as ultimate consequences [39,47]. Therefore, a look at cell viability and proliferation is paramount to data interpretation. However, due to the constraints of the test system, viability data could only be gathered at the end of the trial period in order to minimize disturbances to the cell populations and the model. No significant effects of the PFA on the viability of either cell type could be observed in the absence of a ConA stimulus. The WST-1 cell proliferation and viability assay is a useful tool to measure mitochondrially, and therefore metabolically, active cells [48], especially with PBMC stimulated by the powerful activator ConA [49,50,51]. No significant effects of the PFA treatments on the viability of ConA-stimulated cells compared with the ConA control were observed. This indicates that the observed barrier protective effects were not caused by reduced viability or proliferation of PBMC due to PFA treatment. Similarly, PFA treatments of IPEC-J2 with or without ConA stimulation did not negatively affect viability compared with the respective controls. The absence of negative effects on cell viability furthermore indicates that the cell populations were not negatively affected by nutrient depletion or cellular damage and ageing. However, decreasing barrier protective effects of applied PFAs after the optimum at 48 h may indicate that timely replenishment of fresh media and test compounds may be necessary to keep up barrier protective effects.

Both PFA extracts showed effects dependent on the application site (apical or basolateral compartment), which is in line with previously reported results of some of their major active compounds [40]. Disrupted barrier integrity was successfully counteracted or mitigated, especially due to the basolateral application of both PFAs of interest. This indicates that directly treating the blood lymphocytes, and their inflammation-related signaling pathways, on the systemic side has a bigger influence on overall barrier integrity in this in vitro co-culture model than directly treating the seemingly mainly affected epithelial cell layer on the apical side. This seems to corroborate our assumption that barrier integrity is mainly influenced by the systemic side, e.g., by intercellular messaging and signaling between blood and immune cells, as well as intestinal epithelial cells. For example, Schott et al. reported increased expression of inflammatory cytokines IL-12 and IFN-γ due to stimulation of PBMC with ConA [40]. Similarly, functional cell communication and signaling, also involving the luminal microbiota, is key in a healthy, functional gut in vivo [52]. Additionally, the effects of apical treatment are likely dependent on the transition of active phytochemical compounds to the basolateral side to unfold the beneficial effects of the active compounds on PBMC. Optimum performance after 48 h of basolateral treatment may therefore indicate positive barrier protective effects of the prolonged presence, and continuous circulation of the active compounds in the animal’s blood. In future in vitro trials, this may be achieved by refreshing media and treatments in regular intervals, which was not performed in the present study, in order to reduce the risk of contamination and avoid disturbance of PBMC populations in the basolateral suspension culture. Based on these in vitro findings, continuous inclusion of the tested PFAs in the feed and uptake by live animals is also likely to ensure the constant presence of active compounds in the animals’ systemic circulation, which is necessary to maximize these beneficial observed effects.

### 3.2. Contribution of Phytogenic Components to Observed Effects

The two PFA mixtures investigated in this study are very similar in their main phytochemical components, but at the same time differ in their composition and the origin of contained compounds. All these compounds, especially those known to have effects on barrier integrity, intestinal gut health, and inflammation are of increased importance in the context of this study, as one of the aims was to determine if the observed barrier protective effects can be explained by their presence in the tested PFA. Indeed, significant beneficial effects of licorice extract and oregano oil (containing 60–75% carvacrol) were found for 48 and 72 h after barrier disruption, respectively, using the same test model described in this study [40]. In both cases, barrier protective effects were observed after basolateral, but not after apical addition, showing similar results to the PFAs D-DC1 and D-DC2 investigated here. While both PFAs contain pure carvacrol, D-DC2 additionally contains natural oregano essential oil, containing both carvacrol and to a lesser degree thymol. Carvacrol is the main phytochemical of oregano oil and is therefore likely contributing to its barrier protective effects, and by extension also to the effects of both tested PFAs. Liquorice extract is featured in D-DC2, but not in D-DC1, and therefore may only contribute to the effects of D-DC2. In another study, a PFA that was very similar to D-DC2 containing licorice extract, L-menthol, methyl salicylate, oregano essential oil, as well as a plant powder mix consisting of gentian, angelica root, and cinnamon, was reported to show anti-inflammatory properties in an IPEC-J2 model [44], while the same study also showed antioxidative properties of the contained grape seed extract. These results gave rise to studies in more complex gut models. Hypothetically, barrier protection described in the present study was mediated by the test substance’s anti-inflammatory effects on PBMC populations. The same plant powder mix as used in the current study, containing ground gentian root, angelica root, and cinnamon bark, increased the speed of barrier function recovery after a calcium switch in an IPEC-J2 barrier model, as did licorice and angelica root extracts alone [31], indicating beneficial effects of these plant-based extracts on (gut) barrier function and a likely contribution of the plant powder mix to the performance of D-DC1 and D-DC2. A blend of thymol and cinnamaldehyde showed promising effects in an IPEC-J2 barrier model following pre-incubation for 24 h before the application of a lipopolysaccharide (LPS) challenge simulating inflammation. Barrier integrity was increased and epithelial cell regeneration was enhanced [22]. Studies on mint oils, containing for example L-menthol, on barrier integrity of IPEC-J2, revealed inhibition of inflammation-related cytokine secretion by LPS-challenged porcine alveolar macrophages [25], hinting at possible similar effects on PBMC isolated from porcine blood. Positive effects of carvacrol and thymol on barrier integrity were also reported using a *Salmonella*-challenged Caco-2 model [53], and a combination of thymol and cinnamaldehyde, the major active compound of cinnamon, had beneficial effects on tight junction barrier integrity in a Caco-2 model as well [54]. *In vivo*, blends of carvacrol and thymol have also been reported to support intestinal barrier integrity, e.g., by reducing oxidative stress and modulating the jejunal microbiome of piglets after weaning [9]. PFA mixtures containing carvacrol and thymol have been reported to improve intestinal morphology, immune response, and the expression of tight junction proteins important to intestinal barrier integrity in *E.coli*-challenged weaned piglets [20]. Oregano essential oil, containing both carvacrol and thymol, has been linked to improved intestinal barrier integrity and beneficial effects on growth performance [10,55]. Effects of licorice on the immune function of swine, investigated in the peripheral blood and mucosal tissue after dietary supplementation, have been reported [45], and positive effects of licorice feed supplements have been described for weaned piglets, reducing diarrhea, improving performance, intestinal morphology, and barrier and immune function [46]. Recent in vivo studies with grape extracts as a dietary additive for piglets undergoing weaning have shown promising results regarding intestinal integrity and morphology [16], as well as apparent total tract digestibility when compared with an antibiotic [15]. Positive effects of carvacrol and thymol supplementation have been reported in other livestock such as broiler chicken, where intestinal integrity of Clostridium-challenged chicken was improved, gut lesions alleviated, and effects of necrotic enteritis were reduced [56,57].

In addition to the described effects of the contained substances on areas directly related to the study at hand, i.e., barrier integrity and intestinal health, most of these compounds and substances also feature various other beneficial properties that make them suitable candidates for inclusion in PFAs. Carvacrol, like its structural isomer thymol, is a monoterpenoid phenol usually found in oregano essential oil, and is a main constituent of both PFA mixtures. Both phenols and the essential oil have been reported extensively to provide numerous additional beneficial effects as feed additives in animal nutrition, for example antimicrobial [7,21,53,58,59,60,61] and antioxidative properties [59,61], or positive effects on intestinal health parameters [56,57], both in vitro and in vivo [7,11,12,21,62]. Another major component of both extracts is L-menthol, structurally a monoterpenoid that can be found naturally in peppermint oil. Menthol and peppermint oil have been reported to offer beneficial effects in animal nutrition, for example antibacterial ones, as well as effects on feed intake and conversion, e.g., in chicken [21,63]. Methyl salicylate, a benzoate ester derived biosynthetically from salicylic acid, is also a prominent component of both mixtures. Similar to acetylsalicylate compounds and products (“aspirin”), anti-inflammatory properties have been reported for methyl esters of salicylic acid and glycosides thereof [64]. Both PFAs also feature further plant-based additives and their active components with additional reported beneficial properties, e.g., a complex plant powder mix, including cinnamon bark, and its active component cinnamaldehyde [5,7,11,21,24,65], gentian root, grape seed and skin extract [66,67], and angelica root. Licorice extract, included only in D-DC2, has also been reported as a feed additive with various beneficial properties, e.g., on performance, intestinal health, and the immune system [12,68].

As described above, for many of the substances and compounds contained in D-DC1 and D-DC2, effects in line with the observed results of this study have been reported in vitro and in vivo, and there is strong evidence that they are major contributors to the observed effects. These effects may include both direct modes of action, but also synergistic effects of the combined compounds contributing to the overall effect. A major aim of this study was to investigate whether there would be different effects of the two tested PFAs. In our study, a PFA based mainly on pure single chemical substances (D-DC1) was equivalent to one based mainly on natural substances with a complex composition, such as essential oils and extracts (D-DC2) with regard to barrier protective properties. Comparable efficacy may be assumed when used as feed additives in vivo. Naturally, this is just an indication from the in vitro results described above, that requires further investigation and only covers one specific area of effects.

### 3.3. Outlook and Future Use of the Co-Culture Model

In the present study, PBMCs isolated from the blood of different pigs were used in the four independent trials. The inclusion of these different PBMC populations in the separate trials contributed to overall data variation, as significant effects of the individual trials were observed in the model. However, reproducible and significant beneficial effects of the extracts on barrier integrity were observed, even though unknown biological variation was deliberately introduced to and was part of the trial, due to the use of PBMC populations from different pigs. This finding is interesting when trying to link in vitro data with possible in vivo effects. In our opinion, it further enhances the value of the test model, as well as collected data, as it also accounts for differences between individual, randomly chosen animals, with unknown status regarding factors such as genetics, rearing and feeding, as well as health and immune status. Natural variation between used PBMC populations makes the model more lifelike than test systems using only commercial, often immortalized, cell lines, where similar effects on and of the cells should always be expected. The results described above also lend further importance to our postulation that regular incorporation of co-culture models in in vitro testing is a powerful tool for scientific research, as well as commercial product development. Co-culture models offer a multitude of improvements over models with one cell type and additional parameters that can be investigated, which in turn makes increased resource input and needed effort worthwhile. Indeed, the field of intestinal epithelial models is increasingly shifting from using singular cell lines to more intricate and lifelike models to better approximate in vivo conditions in vitro [29]. While the in vitro study at hand focused on investigating and comparing barrier protective effects of experimental phytogenic feed additives, additional parameters of interest can be used to improve our understanding of test substances and cellular behavior. These parameters include gene expression profiles, cytokine expression and intercellular signaling, the bioavailability of active compounds and passage through the gut barrier, as well as anti-inflammatory and antioxidative properties. The potential of these various additional parameters, in combination with the advantages of introducing ex vivo biological variation inherent to PBMC isolated from the blood to in vitro testing, makes for exciting future prospects and experimental possibilities.

## 4. Materials and Methods

### 4.1. Routine Maintenance of IPEC-J2 Cultures

Intestinal porcine epithelial cells (IPEC-J2; ACC 701, Leibniz Institute DSMZ—German Collection of Microorganisms and Cell Culture, Braunschweig, Germany) were cultivated and routinely maintained using DMEM/F12 (1:1) without L-glutamine (Pan Biotech, Aidenbach, Germany), adjusted to 2.4 g/L NaHCO_3_ (Pan Biotech) and supplemented with 1% insulin–transferrin–selenium ITS (Gibco/Life Technologies, Thermo Fisher Scientific, Vienna Austria), 2.5 mM Glutamax (Gibco/Life Technologies, Thermo Fisher Scientific), 5 ng/mL epidermal growth factor (Corning Inc, Corning, USA), and 16 mM HEPES (Merck/Sigma Aldrich, Vienna, Austria). The medium was further supplemented with 10% heat-inactivated (30 min at 56 °C) fetal bovine serum FBS (Gibco/Life Technologies, Thermo Fisher Scientific) and 1% penicillin–streptomycin (Merck/Sigma Aldrich) directly before use. Cultures were incubated at 39 °C and 10% CO_2_ under a humidified atmosphere (CO_2_ incubator, Binder, Tuttlingen, Germany) and sub-cultivated upon exceeding 90% confluency in the cultivation vessels. Routine testing for mycoplasma contaminations was conducted by PCR (Venor^®^ GEM Classic Mycoplasma Detection Kit, Minerva Biolabs, Berlin, Germany).

### 4.2. Blood Sampling and Isolation of PBMC

PBMC were isolated from porcine blood. Whole blood was sourced from a local slaughterhouse. Pigs, at approximately 6.5 months of age, were chosen randomly and regardless of their gender (female and castrated males). Blood was collected from the pigs’ jugular vein during the exsanguination part of the slaughter. All aspects of the slaughter procedure followed the guidelines and regulations laid down in the Austrian animal welfare legislation (Animal Protection Act—TSchG) [69]. Whole blood was collected in centrifugation tubes containing EDTA (120 mg/mL, Merck/Sigma Aldrich) to prevent coagulation. PBMC were isolated from the porcine blood samples by Ficoll-Paque Plus (GE Healthcare, Chicago, USA) density gradient centrifugation, and either used directly after resuspending the pellet in PBMC cultivation medium, or preserved in cryo-conservation medium for storage at −80 °C until use. PBMC cultivation medium was prepared similarly to the IPEC-J2 cultivation medium, with FBS supplementation reduced to 5% heat-inactivated FBS.

### 4.3. Co-Culture Model of the Piglet Gut

**Figure 4:**
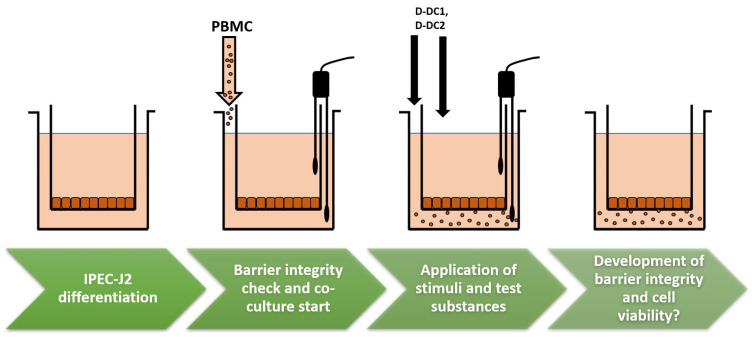
Schematic drawing of the Indirect co-culture model.

Based on the co-culture model of the piglet gut described by Schott et al. [40], IPEC-J2 were seeded in 12-well plates with transwell inserts (Corning Inc.), i.e., in the “apical” inner compartment of the inserts at a seeding density of 1 × 10^5^ cells/well. Cells were allowed to grow and differentiate for seven days, with media change in apical and basolateral compartments every second day (see model schematic in Figure 4). At the end of the differentiation phase, confluent growth in the inserts was checked microscopically and intestinal epithelial barrier integrity was assessed using TEER [kOhm*cm^2^] measurement. TEER was measured with a volt-/ohmmeter (Millicell ERS, Millipore, Burlington, USA) and an adjustable electrode set (MERSSTX03 Millicell ERS Adjustable Electrode Set, Millipore) under sterile conditions (schematic, Figure 4). Upon reaching a minimum TEER of 6 kOhm*cm^2^ on the eighth day after seeding IPEC-J2, the medium was changed from IPEC-J2 cultivation medium to PBMC cultivation medium, and PBMC were seeded to the basolateral compartment at a seeding density of 3*10^6^ cells/well. Plates were further incubated at 39 °C and 10% CO_2_ for 24 h prior to the application of stimulus and treatments to the test wells. Barrier integrity was checked every 24 h after stimulus application (t = 24, 48, and 72 h) and cell viability was assessed following the last measurement. For evaluation, absolute TEER values were assessed statistically, and relative TEER values were calculated using the CC or ConA control groups as reference points (=100%). A total of four separate trials were carried out, each consisting of two trial plates seeded with cells originating from the same culture. Treatments were performed in duplicates on two plates originating from the same culture flask for each independent trial. For co-cultivation, PBMC isolated from the blood of different pigs were used, avoiding cross-contamination and mixing of different PBMC populations.

### 4.4. Stimulation of the Co-Culture Model

Intestinal inflammation and compromised barrier integrity were simulated in vitro by the direct addition of the plant lectin concanavalin A (ConA, Merck/Sigma Aldrich). A final ConA concentration of 1.25 µg/mL was applied to the basolateral compartment of the test system after 24 h of indirect co-cultivation of IPEC-J2 and PBMC. ConA was used to directly activate the PBMC, and create a challenge inducing a deterioration of the intestinal gut barrier integrity of IPEC-J2 growing in the apical compartment. CC and ConA controls were included on all test plates as reference points to compare relative barrier integrity at different time points post-stimulation. Test extracts were applied to the test system at 150 µg/mL, a concentration derived from the cell viability pre-trials with PBMC (Appendix A). Extracts were applied to either the apical or basolateral compartments, with or without simultaneous ConA stimulus to the basolateral compartment. The test products were provided by BIOMIN Phytogenics GmbH (part of DSM) and had a similar composition: encapsulated volatile oil mixtures, extracts, and plant powders. Digestarom^®^ DC 2 (D-DC2) features a higher proportion of natural essential oils than Digestarom^®^ DC 1 (D-DC1), by the inclusion of oregano oil from leaves of *Origanum vulgare* L., while both products contain pure L-menthol, carvacrol, and methyl salicylate. Both volatile oil mixtures are characterized by carvacrol as their main component, accumulating to 85–89 g/kg product. Both products contain grape extract (*Vitis vinifera* L.) and a mixture of plant powders from cinnamon (*Cinnamomum cassia* (L.) D.Don.) bark, gentian root (*Gentiana lutea* L.), and angelica root (*Angelica archangelica* L.), while D-DC2 additionally contains licorice root extract (from *Glycyrrhiza glabra*). Test extracts were prepared freshly before application by extraction of 1 g sample in 10 mL EtOH (70%, VWR), on a shaker (IKA) for 1 h. Solutions were spun down briefly, and supernatants were sterile-filtered (Filtropur S 0.2 µm, Sarstedt, Inc., Nümbrecht, Germany) before preparation of dilutions (with cultivation medium) and application to the test system.

### 4.5. Cell Viability Testing and Supernatant Samples

Pre-trials were carried out in order to determine suitable non-cytotoxic extract test concentrations. PBMC were seeded to 96-well plates (Eppendorf, Hamburg, Germany) at a seeding density of 5*10^5^ cells/well, and incubated at 39 °C and 10% CO2 for 24 h before application of the test substances. Product extracts were diluted with PBMC cultivation medium and spiked to the test wells resulting in test concentrations of 75, 150, 300, and 600 µg/mL. CC was included, as well as a solvent control, and substance blanks of the respective test concentrations in the absence of PBMC. All treatments were applied in duplicates on the plates. Three separate trials were carried out, using PBMC isolated from the blood of three different pigs. After 24 h of incubation with the stimuli, the WST-1 cell proliferation/cytotoxicity test was carried out (ready-to-use kit, Roche, Basel, Switzerland), and evaluated based on the manufacturer’s instructions. In the main challenge trial, the effects of the PBMC activator ConA and/or applied product extracts on the PBMC populations, as well as the IPEC-J2 layer in the transwell inserts were assessed 72 h after the application of stimuli. The viability of IPEC-J2 was assessed directly in the transwell inserts using the neutral-red (NR) uptake assay (TOX4-1 Kit, Merck/Sigma Aldrich). To assess the viability of PBMC, cells in the basolateral compartment were gently resuspended and suspension aliquots were assayed in 96-well plates using the WST-1 cell proliferation test (Roche), as described above. The viability of cells according to NR or WST-1 was evaluated relative to the unstimulated CC and ConA stimulated control.

### 4.6. Statistics

GraphPad Prism 9 (Version 9.1.0 (221), GraphPad Software, Inc, Boston, USA.) was used for statistical analysis, graphs, and diagrams. All data sets were tested for normal distribution, assessing QQ-plots and an array of normality tests (Shapiro–Wilk, D’Agostino Pearson, Anderson–Darling, and Kolmogorov–Smirnov) as a pre-requisite of testing. Normally distributed barrier integrity data (TEER) were further analyzed by repeated measures two-way ANOVA for the three measurement time points, using Dunnett’s multiple comparisons test to compare treatment groups with both stimulated (ConA) and unstimulated (CC) control groups. Cell viability assays were assessed by comparing relative cell viability values to both ConA stimulated or unstimulated control groups, using ordinary one-way ANOVA and Dunnett’s multiple comparisons test. Data sets not normally distributed were further analyzed using the nonparametric Kruskal–Wallis test to compare the means of individual trials. In all cases, differences with *p* < 0.05 were considered statistically significant and marked accordingly in the Results section. Non-significant *p*-values below 0.1 are described as a trend in this study.

## Figures and Tables

**Table 1:** Effects of D-DC1 and D-DC2 extracts (applied at 150 µg/mL) on the co-culture model of the piglet gut. TEER [kOhm*cm^2^] was observed over a 72 h period following stimulation and relative TEER values were calculated compared with the unstimulated CC and the ConA stimulation control. Effects of treatments were compared with both stimulated ConA and unstimulated CC groups for apical (=api) and basolateral (=baso) addition. Relative changes compared with the ConA control are indicated for extract plus ConA treatment groups. Values represent the means of 4 independent experiments (n = 4), as visualized in Figure 2. Statistically significant results, based on absolute TEER values, are indicated by a superscript (^a^ significant difference compared with CC, *p* < 0.05; ^b^ significant difference compared with ConA, *p* < 0.05; ^c^ significant difference compared with both control groups, *p* < 0.05), and *p*-values indicated for comparisons with both CC and ConA. A bold script is used to indicate statistical significance compared with the respective control used for the calculation of the relative TEER value in %. Results for statistical analysis of variation and interactions of row and column factors are included below.

				Extract D-DC1				Extract D-DC2			
		CC	ConA	api	baso	api + ConA	baso + ConA	api	baso	api + ConA	baso + ConA
24 h	TEER [kOhm*cm^2^]	12.73 ^b^	10.52 ^a^	12.49 ^b^	12.28 ^b^	11.08	11.19	12.71 ^b^	12.33 ^b^	10.66 ^a^	11.31
	% of CC	100.0	**82.7**	98.3	96.6	87.2	88.1	99.9	97.0	**83.9**	88.8
	*p*-value vs. CC	-	**0.0265**	0.9943	0.9017	0.0807	0.1164	0.9999	0.9264	**0.0347**	0.2206
	% of ConA	**121.0**	100.0	**118.8**	**116.8**	105.4	106.4	**120.9**	**117.2**	101.4	107.5
	*p*-value vs. ConA	**0.0265**	-	**0.0093**	**0.0185**	0.5124	0.5497	**0.0180**	**0.0139**	0.9994	0.5787
48 h	TEER [kOhm*cm^2^]	12.63 ^b^	9.18 ^a^	12.29 ^b^	12.11 ^b^	10.03 ^a^	10.33 ^c^	12.46 ^b^	11.99 ^b^	9.66 ^a^	10.31 ^c^
	% of CC	100.0	**72.7**	97.3	95.9	**79.5**	**81.8**	98.6	94.9	**76.5**	**81.7**
	*p*-value vs. CC	-	**0.0001**	0.8891	0.6587	**0.0004**	**0.0007**	0.9940	0.7701	**0.0002**	**0.0010**
	% of ConA	**137.6**	100.0	**134.0**	**132.0**	109.4	**112.6**	**135.8**	**130.7**	105.3	**112.4**
	*p*-value vs. ConA	**0.0001**	-	**0.0017**	**0.0035**	0.0507	**0.0082**	**0.0007**	**0.0301**	0.2197	**0.0046**
72 h	TEER [kOhm*cm^2^]	13.05 ^b^	9.10 ^a^	12.67 ^b^	12.43 ^b^	9.85 ^a^	9.98 ^a^	12.83 ^b^	12.29 ^b^	9.24 ^a^	9.81 ^a^
	% of CC	100.0	**69.8**	97.1	95.2	**75.6**	**76.5**	98.3	94.1	**70.9**	**75.2**
	*p*-value vs. CC	-	**0.0001**	0.8359	0.2807	**0.0002**	**0.0005**	0.9625	0.6242	**0.0008**	**0.0002**
	% of ConA	**143.4**	100.0	**139.3**	**136.6**	108.3	109.7	**141.0**	**135.1**	101.6	107.8
	*p*-value vs. ConA	**0.0001**	-	**0.0004**	**0.0002**	0.1787	0.1014	**0.0001**	**0.0115**	0.9849	0.1961

**Figure A1:**
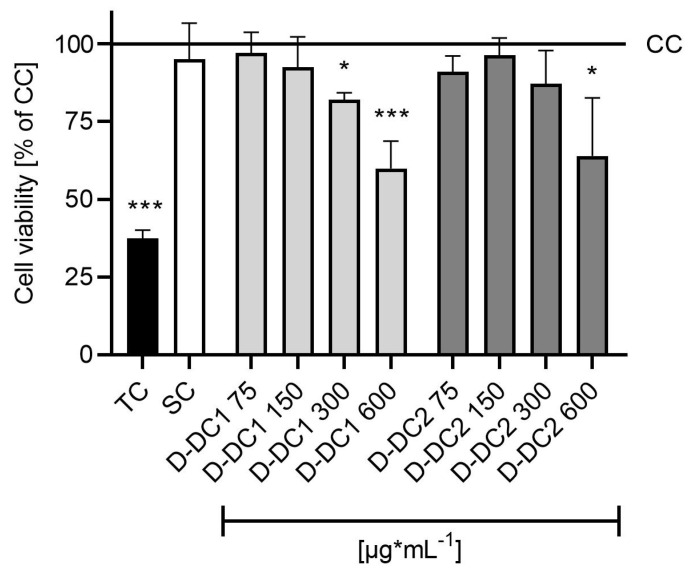
Pre-trial to determine the effects of extracts D-DC1 and D-DC2 on the viability of PBMC over a concentration range. Product extracts were tested at 75–600 µg/mL. Cell viability was determined using the WST-1 cell proliferation assay. Bars and error bars represent means and standard deviations from 3 independent trials, using PBMC isolated from the blood of 3 different pigs (n = 3). Untreated CC was set to 100% and is represented as a solid line in the graph. A toxicity control (TC) with cytotoxic effects, as well as a solvent control (SC) were included. Statistically significant differences compared with the CC are indicated by asterisks (* *p* < 0.05, *** *p* < 0.001).

## Data Availability

Not applicable.

## Appendix A. Cytotoxicity Pre-Trial

Viability testing was carried out using the WST-1 cell proliferation reagent, to assess the effects of the product extracts on cellular viability and proliferation of PBMC isolated from the blood of three different pigs (Figure A1). D-DC1 reduced viability significantly compared with untreated CC by 18.0 (*p* < 0.05) and 40.1% (*p* < 0.001), when applied at 300 and 600 µg/mL, respectively. D-DC2 significantly reduced viability by 36.1% (*p* < 0.05), when applied at 600 µg/mL. No significant effects were observed for other treatment concentrations. Treatment of cells with 150 µg/mL did not lead to a reduction in cellular viability for both extracts (*p* > 0.05), and was therefore chosen as a suitable concentration for testing in the co-culture model.

## Appendix B. Results of Repeated Measures Two-Way ANOVA

**Table A1:** Results of repeated measures two-way ANOVA, regarding the source of variation and contribution of row and column effects to overall data variation. The percentage of total variation caused by the respective factors is given, as well as *p*-Values.

Source of Variation	% of Total Variation	*p*-Value
Treatment × time	5.390	<0.0001
Treatment	79.09	<0.0001
Time	4.592	<0.0001
Trial	6.039	0.0014
